# GraphCrunch: A tool for large network analyses

**DOI:** 10.1186/1471-2105-9-70

**Published:** 2008-01-30

**Authors:** Tijana Milenković, Jason Lai, Nataša Pržulj

**Affiliations:** 1 Department of Computer Science, University of California, Irvine, CA 92697-3435, USA

## Abstract

**Background:**

The recent explosion in biological and other real-world network data has created the need for improved tools for large network analyses. In addition to well established *global *network properties, several new mathematical techniques for analyzing *local *structural properties of large networks have been developed. Small over-represented subgraphs, called network *motifs*, have been introduced to identify simple building blocks of complex networks. Small induced subgraphs, called *graphlets*, have been used to develop "network signatures" that summarize network topologies. Based on these network signatures, two new highly sensitive measures of network local structural similarities were designed: the *relative graphlet frequency distance *(*RGF-distance*) and the *graphlet degree distribution agreement *(*GDD-agreement*).

Finding adequate null-models for biological networks is important in many research domains. Network properties are used to assess the fit of network models to the data. Various network models have been proposed. To date, there does not exist a software tool that measures the above mentioned local network properties. Moreover, none of the existing tools compare real-world networks against a series of network models with respect to these local as well as a multitude of global network properties.

**Results:**

Thus, we introduce GraphCrunch, a software tool that finds well-fitting network models by comparing large real-world networks against random graph models according to various network structural similarity measures. It has unique capabilities of finding computationally expensive RGF-distance and GDD-agreement measures. In addition, it computes several standard global network measures and thus supports the largest variety of network measures thus far. Also, it is the first software tool that compares real-world networks against a series of network models and that has built-in parallel computing capabilities allowing for a user specified list of machines on which to perform compute intensive searches for local network properties. Furthermore, GraphCrunch is easily extendible to include additional network measures and models.

**Conclusion:**

GraphCrunch is a software tool that implements the latest research on biological network models and properties: it compares real-world networks against a series of random graph models with respect to a multitude of local and global network properties. We present GraphCrunch as a comprehensive, parallelizable, and easily extendible software tool for analyzing and modeling large biological networks. The software is open-source and freely available at . It runs under Linux, MacOS, and Windows Cygwin. In addition, it has an easy to use on-line web user interface that is available from the above web page.

## Background

### Motivation

The recent technological advances in experimental biology have yielded large amounts of biological network data. Many other real-world phenomena have also been described in terms of large networks (also called graphs), such as various types of social and technological networks. Thus, understanding these complex phenomena has become an important scientific problem that has lead to intensive research in network modeling and analyses.

Analogous to genetic sequence comparison, comparing large cellular networks will revolutionize biological understanding. However, comparing large networks is computationally infeasible due to NP-completeness of the underlying subgraph isomorphism problem [1]. Note that even if the subgraph isomorphism was feasible, it would not find a practical application in biological network comparisons, since biological networks are extremely unlikely to be isomorphic. Thus, large network comparisons rely on heuristics, commonly called *network parameters *or *properties*. These properties are roughly categorized into *global *and *local*. The most widely used global properties are the *degree distribution*, the *clustering coefficient*, the *clustering spectrum*, the *average diameter *and the *spectrum of shortest path lengths *[2] (defined in Section "Global network properties"). Local properties include *network motifs*, small over-represented subgraphs [3-5], and two measures based on *graphlets*, small induced subgraphs of large networks: the *relative graphlet frequency distance *(*RGF-distance*), which compares the frequencies of the appearance of graphlets in two networks [6], and the *graphlet degree distribution agreement *(*GDD-agreement*), which is a graphlet-based generalization of the degree distribution [7] (details are given in Section "Local network properties").

Various network models have been proposed for real-world networks. Starting with Erdös-Rényi random graphs [8], network models have progressed through a series of versions designed to match certain properties of real-world networks. Examples include random graphs that match the degree distribution of the data [9], network growth models that produce networks with scale-free degree distributions [10] or small network diameters [11], geometric random graphs [12], or networks that reproduce some biological and topological properties of real biological networks (e.g., stickiness model [13]).

Currently, there does not exist a software tool that measures all of the above mentioned global and local network properties. Computing global network properties is computationally and conceptually easy and various software tools are available for this purpose. However, none of them have built-in capabilities to compare real-world networks against a series of network models, based on these properties. The computational challenge is in finding local properties. Currently available software packages focus on searching for network motifs [14-16]. Thus far, there does not exist a publicly available, open-source software tool that computes local properties other than network motifs. For this reason, GraphCrunch primarily focuses on graphlets and has the unique capabilities of computing RGD-distance and GDD-agreement measures of local network structure. We introduce GraphCrunch as a comprehensive, parallelizable, and easily extendible open-source software tool for analyzing and modeling of large real-world networks.

### Methods

GraphCrunch automates the process of generating random networks drawn from user specified random graph models and evaluating the fit of the network models to a real-world network with respect to global and local network properties. In a single command, GraphCrunch performs all of the following tasks: 1) computes user specified global and local properties of an input real-world network, 2) creates a user specified number of random networks belonging to user specified random graph models, 3) compares how closely each model network reproduces a range of global and local properties (specified in point 1 above) of the real-world network, and 4) produces the statistics of network property similarities between the data and the model networks. The properties and models currently supported by GraphCrunch are presented in Tables 1 and 2, respectively. GraphCrunch is easily extendible to include additional network properties and models (see Section "Implementation").

**Table 1:** Network properties currently supported by GraphCrunch.

*Global Properties*:
Degree distribution
Clustering coefficient
Clustering spectrum
Average diameter
Spectrum of shortest path lengths

*Local Properties*:

Relative graphlet frequency distance (RGF-distance)
Graphlet degree distribution agreement (GDD-agreement)

**Table 2:** Network models currently supported by GraphCrunch.

*Models*:
Erdös-Rényi random graphs
Random graphs with the same degree distribution as the data
Scale-free Barabási-Albert model graphs
*N*-dimensional geometric random graphs
Stickiness model graphs

#### Network models

GraphCrunch currently supports five different types of random graph models: (1) Erdös-Rényi random graphs (henceforth denoted by "ER") [8]; (2) random graphs with the same degree distribution as the data (henceforth denoted by "ER-DD") [9]; (3) Barabási-Albert type scale-free networks (henceforth denoted by "SF-BA") [10]; (4) *n*-dimensional geometric random graphs for all positive integers *n *(henceforth denoted by "GEO-*n*D") [12]; and (5) stickiness model networks (henceforth denoted by "STICKY") [13].

All generated model networks have the number of nodes and edges within 1% of those in the real-world networks. The model network generators are implemented as follows. Erdös-Rényi random graphs are generated by using the LEDA [17] implementation of *G*_*n*,*m*_, a random graph *G *with *n *nodes and *m *edges. Random graphs with the same degree distribution as the data are generated by using the "stubs method" (see section IV.B.1 of [2] for details): the number of "stubs" (to be filled by edges) is assigned to each node in the model network according to the degree distribution of the real-world network; edges are created between pairs of nodes picked at random; after an edge is created, the number of "stubs" left available at the corresponding "end-nodes" of the edge is decreased by one. Scale-free networks are generated by using the Barabási-Albert preferential attachment model [10]. Geometric random graphs are defined as follows: nodes correspond to uniformly randomly distributed points in a metric space and edges are created between pairs of nodes if the corresponding points are close enough in the metric space according to some distance norm. A variant of geometric random graphs supported by GraphCrunch uses *n*-dimensional Euclidean boxes and the Euclidean distance norm. The default is set to be the 3-dimensional Euclidean space ("GEO-3D"), but user specified dimensions are also supported. Finally, "stickiness network model" is based on stickiness indices, numbers that summarize node connectivities and thus also the complexities of binding domains of proteins in protein-protein interaction (PPI) networks (see [13] for details).

#### Local network properties

Due to NP-completeness of the underlying subgraph isomorphism problem, large network comparisons rely on network properties. These heuristics need to encompass large number of constraints, in order to reduce degrees of freedom in which networks being compared can vary. GraphCrunch has the unique capability to compute two highly constraining measures of local structural similarities between two networks: RGF-distance [6] and GDD-agreement [7]. These measures are based on graphlets, small connected non-isomorphic induced subgraphs of large networks [6]. Note that graphlets are different from network motifs since they must be induced subgraphs (motifs are partial subgraphs) and since they do not need to be over-represented in the data when compared with "randomized" networks. An *induced subgraph *of a graph *G *on a subset *S *of nodes of *G *is obtained by taking *S *and all edges of *G *having both end-points in *S*; *partial subgraphs *are obtained by taking *S *and some of the edges of *G *having both end-points in *S*. Since the number of graphlets on *n *nodes increases super-exponentially with *n*, GraphCrunch currently bases its RGF-distance and GDD-agreement computations on 3–5-node graphlets (presented in Figure S1 in Additional file 1).

RGF-distance compares the frequencies of the appearance of all 3–5-node graphlets in two networks (see [6] for details). Note that the networks being compared by GraphCrunch always have the same number of edges, and thus the frequencies of occurrence of the only 1-node graphlet, a node, and the only 2-node graphlet, an edge, are also taken into account by this measure. Thus, RGF-distance encompasses 31 similarity constraints by examining the fit of 31 graphlet frequencies.

GDD-agreement generalizes the notion of the degree distribution to the spectrum of *graphlet degree distributions *(*GDDs*) in the following way [7]. The degree distribution measures the number of nodes of degree *k*, i.e., the number of nodes "touching" *k *edges, for each value of *k*. Note that an edge is the only graphlet with two nodes (graphlet denoted by *G*_0 _in Figure S2 in Additional file 1). GDDs generalize the degree distribution to other graphlets: they measure for each graphlet *G*_*i*_, *i *∈ 0, 1,..., 29, (illustrated in Figure S2 in Additional file 1) the number of nodes "touching" *k *graphlets *G*_*i *_at a particular node. A node at which a graphlet is "touched" is topologically relevant, since it allows us to distinguish between nodes "touching", for example, a copy of graphlet *G*_1 _in Figure S2 in Additional file 1 at an end-node, or at the middle node. This is summarized by *automorphism orbits *(or just *orbits*, for brevity), as illustrated in Figure S2 in Additional file 1: for graphlets *G*_0_, *G*_1_,..., *G*_29_, there are 73 different orbits, numerated from 0 to 72 (see [7] for details). For each orbit *j*, we measure the *j*^*th *^*GDD*, i.e., the distribution of the number of nodes "touching" the corresponding graphlet at orbit *j*. Thus, the degree distribution is the 0^*th *^GDD. The *j*^*th *^*GDD-agreement *compares the *j*^*th *^GDDs of two networks (see [7] for details). The total GDD-agreement between two networks is the arithmetic or the geometric average of the *j*^*th *^GDD-agreements over all *j *(henceforth arithmetic and geometric averages are denoted by "amean" and "gmean", respectively). GDD-agreement is scaled to always be between 0 and 1, where 1 means that two networks are identical with respect to this property. By calculating the fit of each of the 73 GDDs of the networks being compared, GDD-agreement encompasses 73 similarity constraints. Furthermore, each of these 73 constraints enforces a similarity of two distributions, additionally restricting the ways in which the networks being compared can differ. (Note that the degree distribution is only one of these 73 constraints.) Therefore, GDD-agreement is a very strong measure of structural similarity between two networks. Both of the RGF-distance and GDD-agreement measures were used to discover a new, well-fitting, geometric random graph model of PPI networks [6,7]. In Section "A case study", we illustrate the biological importance of choosing a well-fitting network null model of PPI networks.

#### Global network properties

Global network properties currently supported by GraphCrunch are the degree distribution, the average network diameter, the spectrum of shortest path lengths, the average clustering coefficient, and the clustering spectrum. The *degree *of a node is the number of edges incident to the node. The *degree distribution*, *P*(*k*), describes the probability that a node has degree *k*. The smallest number of links that have to be traversed to get from a node *x *to a node *y *in a network is called the *distance *between nodes *x *and *y *and a path through the network that achieves this distance is called the *shortest path *between nodes *x *and *y*. The average of shortest path lengths over all pairs of nodes in a network is called the average *network diameter*. The *spectrum of shortest path lengths *is the distribution of shortest path lengths between all pairs of nodes in a network. The *clustering coefficient *of a node *z *in a network, *C*_*z*_, is defined as the probability that two nodes *x *and *y *which are connected to the node *z *are themselves connected. The average of *C*_*z *_over all nodes *z *of a network is the *clustering coefficient*, *C*, of the network; it measures the tendency of the network to form highly interconnected regions called clusters. The distribution of the average clustering coefficients of all nodes of degree k in a network is the *clustering spectrum*, *C*(*k*).

## Implementation

GraphCrunch can be downloaded from the GraphCrunch home page (see Section "Availability and requirements"). It runs under Linux, MacOS, and Windows Cygwin. GraphCrunch is implemented in C++, Perl, and Bourne Shell scripts. The programs for generating model networks and calculating network properties are implemented using C++ and the LEDA library for combinatorial and geometric computing [17]. Shell scripts collect the output of these programs to create the final results of the analyses, as well as the graphical and tabular representation of the statistics summarizing the results. The programs and the shell scripts are organized in the directories presented in Table S1 in Additional file 1. This organization allows for easy extendibility of the software to include additional network models and properties (see Section 1 in Additional file 1 for details). Furthermore, GraphCrunch has built-in parallel computing capabilities: it distributes its processes over a user specified cluster of machines. Installation details are described in Section 1 in Additional file 1.

### GraphCrunch interfaces

There are three ways of running GraphCrunch: via the *command-line *interface, the *run-dialog *interface, and the *on-line web *interface. Upon installation, a user can choose either the command-line or the run-dialog interface. The command-line interface allows for specifying all of the following in a single command: the real-world network (input graph) to be processed, the random graph models against which the data is to be compared, the number of networks to be generated per random graph model, the network properties and comparisons between the data and the model networks, and the name of the output data file (see Section 2.1 in Additional file 1 for details). The run-dialog interface is available for the Linux and MacOS versions of GraphCrunch. It provides the same functionality as the command-line interface in a perhaps more user-friendly manner: it guides a user through the sequence of screens, step by step, as presented in Section 2.2 in Additional file 1 (also see Figure S3 in Additional file 1). The input and output formats of these two interfaces are similar (described in Sections "Input format" and "Output format and results" below) and they both allow for large-scale scientific computing network analysis and modeling projects. The GraphCrunch on-line web user interface (available from the GraphCrunch webpage) is provided for non-expert users and those with less intensive processing needs; we recommend that first-time users start with on-line GraphCrunch. The on-line GraphCrunch is described in more details in Section 2.3 in Additional file 1 (also see Figures S4, S5, and S6 in Additional file 1).

### Input format

GraphCrunch supports two input graph formats: the LEDA graph format (.*gw*) and the "edge list" format (.*txt*). The specifics of the LEDA graph format are given at the GraphCrunch web page. The edge list format is simply the graph adjacency list, i.e., the list of node pairs (edges of the network) separated by tabs or spaces, with one node pair per line. The current implementation of GraphCrunch deals with undirected, simple (i.e., no loops or multiple edges), unweighted graphs. Thus, for either of the above two formats, GraphCrunch automatically removes all self-loops, multiple edges, and edge directions. Dealing with directed weighted edges is a subject of future work (see Section "Future directions").

### Output format and results

GraphCrunch creates three types of output: the tabular output file, the set of intermediate files, and the visualized output.

The *tabular output *file is a spreadsheet of tab-separated values (.*tsv*) that contains summarized output statistics. An example of a GraphCrunch output file is presented in Table S2 in Additional file 1 (also see Section 2.4 in Additional file 1).

The *set of intermediate files *includes generated model networks corresponding to the input network, in LEDA graph (.*gw*) format, and the files containing the network properties (e.g., clustering spectra, graphlet counts, graphlet degree distributions etc.). The intermediate files allow for additional analyses of the results without performing any additional compute-intensive processing. Also, the tabular output file contains only the statistics of network parameter similarities between the data and the model networks, but not the results from which the statistics were computed – these results are contained in the intermediate files. For example, the GDD-agreement presented in the output file is a single number between 0 and 1 while the intermediate files contain the actual GDDs for each of the 73 orbits. Thus, intermediate files are needed if one needs to analyze a particular GDD. Similar holds for other network properties.

The *visualized output *is a set of files (in .*ps *format) that contain user-friendly graphical interpretations of the results presented in the tabular output file. One graphical file (plot) is created per network property. A plot illustrates the fit of the network models to one or more real-world networks with respect to the given property. Thus, in a single plot, it is possible to simultaneously illustrate the fit of network models to many real-world networks with respect to one property. Examples of plots are presented in Figure S7 in Additional file 1.

Further details about output results are available in Section 2.4 in Additional file 1.

## Results

### A case study

#### Motivation

Since modeling bio-chemical networks is a vibrant research area, we analyze protein-protein interaction (PPI) networks to illustrate functionality of GraphCrunch. In a PPI network, nodes correspond to proteins and undirected edges represent physical interactions between the proteins. The choice of an appropriate null model can have important implications for any graph-based analysis of PPI networks. For example, the use of an adequate null model is vital for structural motif discovery, which requires comparing real-world PPI networks with randomized ones [18,19]. Using an inappropriate network null model may identify as overrepresented (underrepresented) subgraphs that otherwise would not have been identified. Another example is that a good null model can be used to guide biological experiments in a time- and cost-optimal way and thus minimize the costs of interactome detection [20].

Here, we illustrate a way in which GraphCrunch could be used to help answer some of the PPI network modeling questions. The results presented in this section are not intended for suggesting the best-fitting null model for PPI networks, but rather as an illustration of a possible application of GraphCrunch. Some of the questions that one might ask are the following. What is the best-fitting null model for PPI networks? Do all PPI networks belong to the same graph family, or are they categorized into graph subfamilies? How does the incompleteness of the PPI data affect the choice of an appropriate null model? What is the effect of the noise in PPI networks? Can a unique null model (or a model family) be used for motif search in all PPI networks?

#### Analysis

A well-fitting null model should generate graphs that closely resemble the structure of real networks. This closeness in structure is reflected across a wide range of statistical measures. Thus, testing the fit of a model entails comparing model-derived random graphs to real networks according to these measures. The more constraining the measures are, the fewer degrees of freedom exist in which the compared networks can vary. Thus, by using highly constraining measures a better-fitting null model can be found. GraphCrunch supports various global and local measures of network structure. Global network properties, such as the degree distribution, may not be detailed enough to capture the complex topological characteristics of PPI networks. Thus, here we focus on RGF-distance and GDD-agreement that impose a large number of constraints on the networks being compared (as explained in Section "Local network properties").

We analyze PPI networks of the eukaryotic organisms yeast *Saccharomyces cerevisiae*, fruitfly *Drosophila melanogaster*, nematode worm *Caenorhabditis elegans*, and human. To illustrate the process of finding the best-fitting null model for a given real-world network, we first generate three random networks belonging to each of the five random network models currently supported by GraphCrunch. Next, we compare the real-world network with model networks according to RGF-distance and GDD-agreement measures. Finally, we report the statistics of the fit of each of the network models to the real-world network.

##### Completeness of interactomes

First, we analyze higher-confidence ("core") parts of PPI networks of the four organisms mentioned above. We denote by "yeast-core" the yeast high-confidence PPI network described by von Mering et al. [21], consisting of 988 proteins and 2,455 interactions among them. We denote by "fly-core" the fruitfly *D. melanogaster *high-confidence PPI network published by Giot et al. [22], containing 4,602 proteins and 4,637 interactions. We denote by "worm-core" the worm *C. elegans *core PPI network described by Li et al. [23], containing 1,356 proteins and 1,983 interactions. Finally, we denote by "human-core" the human core PPI network described by Stelzl et al. [24], having 363 proteins and 756 interactions.

**Figure 1:**
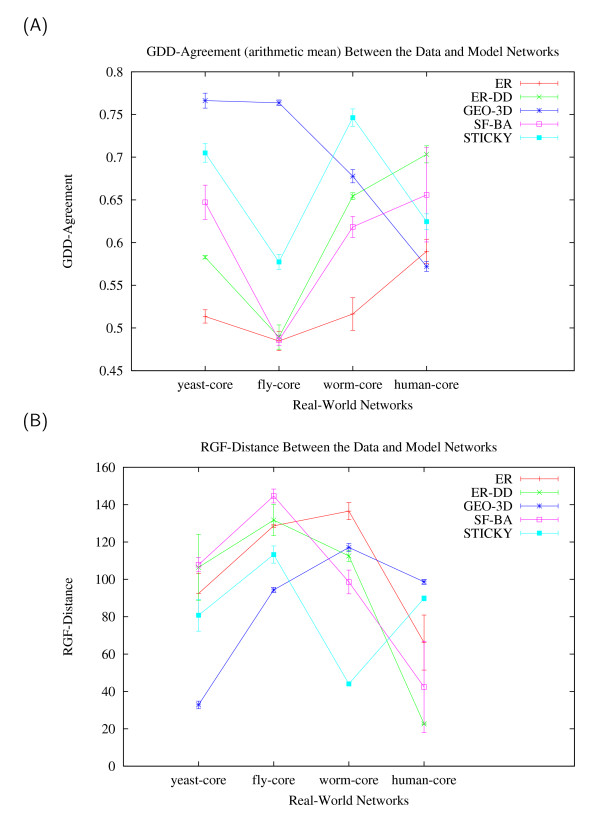
**An illustration of GraphCrunch application to four core PPI networks of different organisms**. The figure shows the fit of five network models (ER, ER-DD, GEO-3D, SF-BA, and STICKY) to four PPI networks (yeast-core, fly-core, worm-core, and human-core) with respect to two networks properties: (A) GDD-agreement, (B) RGF-distance. The larger the GDD-agreement in panel A, the better the fit, and the smaller the RGF-distance in panel B, the better the fit [6,7].

The graphical output of GraphCrunch describing the fit of network models to these four core PPI networks is presented in Figure 1. With respect to both GDD-agreement (Figure 1A) and RGF-distance (Figure 1B), the best-fitting network model for yeast-core and fly-core PPI networks is GEO-3D, the best-fitting null model for worm-core PPI network is STICKY, and the best-fitting null model for human-core PPI network is ER-DD (see Figure 1). Thus, different random graph models provide the best fit to the currently known core PPI networks of different organisms. This might lead to several different conclusions.

It is possible that PPI networks of different organisms belong to different graph families. It is also possible that they belong to the same graph family, but that the current incompleteness of the interactome data for these organisms produces apparent PPI network structural differences [25,26]. Since the two PPI networks of yeast and fruitfly are more extensively studied and thus are more complete, and since they belong to the same graph family, it is possible that the latter interpretation is correct. If we accept this hypothesis, then the improved fit of the two degree-preserving network models (ER-DD and STICKY) to the two less complete PPI networks of worm and human may be attributed to their incompleteness rather than to their true biological structure. Regardless of the reasons for the difference in well-fitting null models of these four PPI networks, a question of whether the same random graph model should be used to identify structural motifs in PPI networks remains.

##### Noise in interactomes

**Figure 2:**
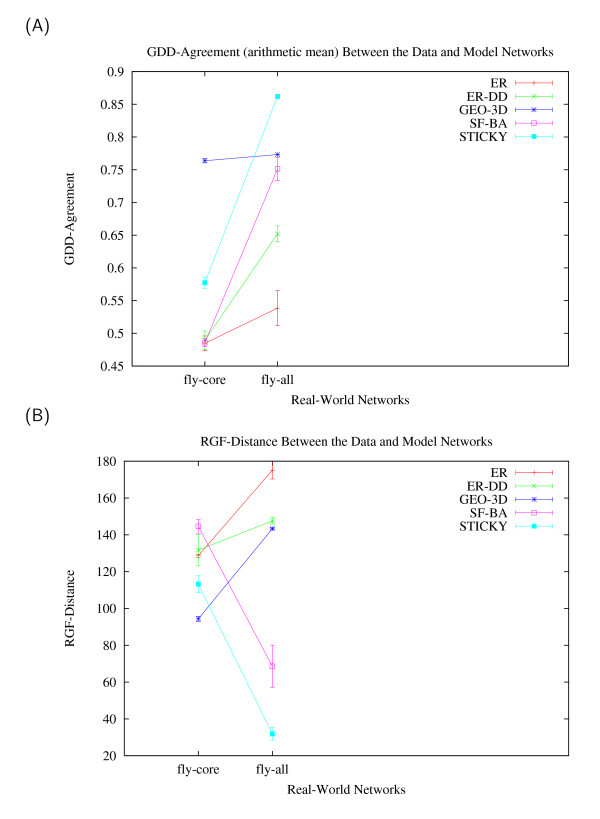
**An illustration of GraphCrunch application to two fruitfly PPI networks of different confidence levels**. The figure shows the fit of five network models (ER, ER-DD, GEO-3D, SF-BA, and STICKY) to two PPI networks (fly-core and fly-all) with respect to two networks properties: (A) GDD-agreement, (B) RGF-distance. The larger the GDD-agreement in panel A, the better the fit, and the smaller the RGF-distance in panel B, the better the fit [6,7].

To address the effect of noise in PPI networks, we analyze the fruitfly *D. melanogaster *core part ("fly-core" PPI network described above) and the entire fruitfly PPI network (denoted by "fly-all", containing 20,007 interactions among 6,985 proteins) as published by Giot et al. [22]. The "fly-all" network has been reported to contain 77% of low-confidence interactions [22], and thus is a very noisy PPI network. The GraphCrunch graphical output illustrating the fit of network models to these two fruitfly PPI networks is presented in Figure 2. With respect to both GDD-agreement (Figure 2A) and RGF-distance (Figure 2B), GEO-3D model provides the best fit to the fly-core PPI network over the other four models, whereas STICKY model provides the best fit to the fly-all PPI network. Thus, this is an example where PPI networks of the same organism, but of different confidence levels, belong to different graph families. This raises a question of whether a higher-confidence, but less complete, or a more noisy, but larger PPI network should be used to draw conclusions about PPI network structure and function.

#### A case study: conclusions

There are many open questions when analyzing and modeling PPI networks. As demonstrated, GraphCrunch is a tool for large network analyses and modeling, capable of finding well-fitting random network models for real-world networks according to various local (illustrated above) and global network properties. It outputs the analysis and modeling results in the form that allows for easy comparisons of different real-world networks. As such, it can be a useful tool in the process of gathering the knowledge necessary to answer many biological questions.

### GraphCrunch performance

To evaluate the performance of GraphCrunch and its behavior with increasing network complexity, we perform the following analyses:

• First, we observe GraphCrunch's running times for input networks with different number of nodes and edges, but with constant edge densities. We determine the edge density |*E*|/|*V*| (where |*V*| is the number of nodes and |*E*| is the number of edges in a network) to be 2.94, by taking the average edge density of five yeast PPI networks. We use the yeast PPI networks since the yeast interactome has been extensively studied and thus is more complete compared to interactomes of other organisms. We find the average edge density of the following yeast PPI networks: the network containing only high-confidence interactions described by von Mering et al. [21], the network containing the top-ranked 11,000 interactions described by von Mering et al. [21], the "core" subset of the yeast PPI network from DIP [27,28], and the entire yeast PPI networks from DIP [27] and MIPS [29] databases downloaded in April 2007. We generate input networks consisting of 100, 500, 1,000, 2,000, 3,000, 4,000, 5,000, 7,500, and 10,000 nodes, and thus with 294, 1,471, 2,941, 5,882, 8,820, 11,764, 14,706, 22,050, and 29,411 edges, respectively, enforcing the same edge density in each of these networks.

• Next, we measure and compare the running times of GraphCrunch for networks with the same number of nodes, but with different edge densities. We observe edge densities smaller, equal to, and larger than the average edge density in yeast PPI networks described above. We generate input networks with 1,000 nodes using edge densities of about 2, 3, 4, 7, and 11, resulting with 2,273, 2,941, 4,167, 7,143, and 11,100 edges amongst 1,000 nodes, respectively.

• Finally, we measure the influence of different properties of input networks on performance of GraphCrunch, by testing as input data networks belonging to different graph models. We use three different graph model generators to create the input networks: ER, GEO-3D, and SF-BA (introduced in Section "Network models"). These input networks differ in the following: GEO-3D model networks have high clustering coefficients and Poisson degree distributions; ER model networks have low clustering coefficients and Poisson degree distributions; and SF-BA model networks have low clustering coefficients and power-law degree distributions. Therefore, we are able to evaluate the effect of the degree distribution and the clustering coefficient measures on the performance of GraphCrunch, by directly comparing running times for input networks drawn from different network models.

We run GraphCrunch once for each of the input networks. We used the following GraphCrunch settings for each run. GraphCrunch compares the input network against all five network models that it currently supports. It generates three random networks per network model and thus, it analyzes 15 model networks per input network. When comparing input and model networks, GraphCrunch computes all network properties that it currently supports.

**Figure 3:**
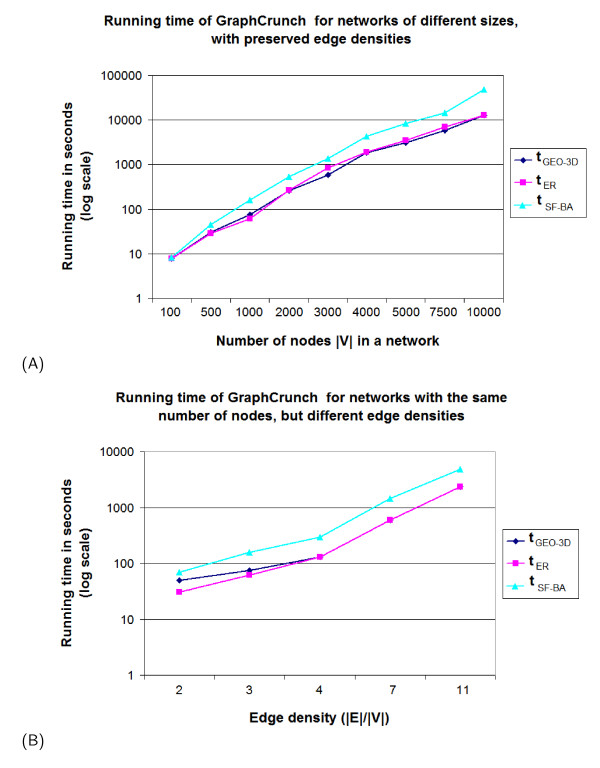
**The running time of GraphCrunch**. The running times *t*_*ER*_, *t*_*GEO*-3*D*_, and *t*_*SF*-*BA *_of GraphCrunch for input networks *G*(|*V*|, |*E*|) (|*V*| being the number of nodes and |*E*| being the number of edges) originating from three different models (ER, GEO-3D, and SF-BA), respectively: (A) the running times for input networks of different sizes, but with constant edge densities; (B) the running times for input networks with the same number of nodes, but different edge densities. We report total number of CPU time, in seconds, that each GraphCrunch run used.

The running times of GraphCrunch for input networks of different sizes, with preserved edge densities, drawn from three different models (ER, GEO, and SF-BA), are presented in Figure 3A (also see Table S3 in Additional file 1). The running times of GraphCrunch for input networks with the same number of nodes, but with different edge densities, drawn from three different models (ER, GEO, and SF-BA), are presented in Figure 3B (also see Table S4 in Additional file 1).

Since GraphCrunch exhaustively finds all graphlets in the input and model networks, the exponential increase in the running time is expected (Figure 3). Note however, that the largest currently available PPI networks have about 6,000 nodes, which coupled with GraphCrunch's parallel computing capabilities gives reasonable processing times (also see Section "Future directions" for planned future speedups). The degree distribution has an influence on the performance of GraphCrunch. Since input scale-free SF-BA networks contain highly connected nodes (hubs), during the exhaustive graphlet enumeration process GraphCrunch severely overcounts graphlets in the vicinity of hubs (of course, the overcounting is corrected after the completion of the exhaustive graphlet enumeration and GraphCrunch returns correct graphlet counts) which degrades its time performance (see [30] for details). Thus, if a large dense network with scale-free degree distribution is being compared to a large number of random graphs mainly belonging to scale-free network models, a user may want to consider distributing the processing over a cluster of machines. Clustering coefficients do not have an influence on the performance of GraphCrunch.

In addition to the analyses described above, we evaluate GraphCrunch's performance against the performances of other network analysis software tools. See Section "Comparison with the existing tools" for details.

### GraphCrunch applications

GraphCrunch is primarily intended for analyzing and modeling of biological networks, although it can be used for other real-world networks as well. GraphCrunch has been applied to and validated through several research projects, some of which have already been published in prestigious refereed journals such as *Science*, *Bioinformatics*, *Journal of the Royal Society Interface*, and *PLoS Computational Biology *[6,7,13,31,32]. For example, GraphCrunch was used for analyzing global network properties of a mammalian TGF*β *signaling network [31]. Also, it was used for computing RGF-distances between several real-world PPI networks and model networks [6], as well as for finding GDD-agreements between PPI and model networks [7]. These studies demonstrated that PPI networks of several eukaryotic organisms are better modelled by geometric random graphs then by scale-free networks [6,7]; this was the first time that geometric random graphs were used to model PPI networks. GraphCrunch was also used to demonstrate the superiority of the fit of the newly introduced stickiness-index based network model of PPI networks over other network models [13]. Furthermore, GraphCrunch was used to compare graphlet frequency distributions of the preferential attachment-based and the duplication-based scale-free model networks that were created by starting with different *seed graphs *and to demonstrate that different seed graphs influence the topologies of the resulting networks [32]. In addition to analyzing and modeling PPI networks described above, GraphCrunch has recently been successfully applied to other types of biological networks such as biomolecular interaction and cellular interaction networks (work in progress).

## Discussion

### Comparison with the existing tools

The network analysis software tools with the functionality similar to the functionality of GraphCrunch include: mfinder [14] with its visualization interface mDraw, MAVisto [15], FANMOD [16], TopNet [33] with its successor tYNA [34], and pajek [35]. While some of these tools compute only local network properties, others that analyze both local and global properties offer fewer functions than GraphCrunch does. The summary of the functions of these tools is presented in Table 3.

**Table 3:** The summary of the functionalities of GraphCrunch and similar purpose software tools. "Yes" and "No" denote that a software package supports or does not support a given function, respectively.

Software tool	Global properties	Local properties	Visualization	Number of network models	Output of model networks	Input graph format
GraphCrunch	Yes	Yes	Yes (Results)	5	Yes	LEDA graph format (.gw); Edge list (.txt)
mfinder	No	Yes	Yes, with mDraw	3	Yes (not by default)	Edge list (.txt)
MAVisto	No	Yes	Yes	1	No	Pajek format (.net) and .gml
FANMOD	No	Yes	Yes	3	No	Edge list (.txt)
tYNA	Yes (limited)	Yes (limited)	Yes	0	No	Edge list (.txt) and .sif
pajek	Yes	Yes (limited)	Yes	2	Yes	Pajek format (.net)

Compared to the tools mentioned above, GraphCrunch provides the largest variety of network properties. The main purpose of mfinder, MAVisto, and FANMOD is motif search; they do not compute global network properties. On the other hand, pajek focuses on global network properties and has very limited local network analysis capabilities; its search for subgraphs is limited to 3–4-node rings. tYNA's global and local network analyses are limited: it calculates the statistics of global network properties and focuses on three network motif types only. Unlike any of these software packages, GraphCrunch uses all of the 2–5-node graphlets for computing its two highly constraining graphlet-based local network properties, GDD-agreement [7] and RGF-distance [6] (described in Section "Local network properties"), along with five standard global properties (described in Section "Global network properties"). We chose to use all 2–5-node graphlets for the following reasons. There are only nine 2–4-node graphlets, so if only these would be used, the complexity of the local network topologies of networks being compared would not be captured. On the other hand, using graphlets with 6 or more nodes would substantially increase the computational complexity. Since we provide GraphCrunch as an open-source software package, users could choose to modify this or any other part of the code to fit their particular needs.

Furthermore, GraphCrunch uses all of these properties for comparing real-world networks against a series of network models. Five network models are currently supported by GraphCrunch. Although Mfinder, FANMOD and pajek offer more than one network model (MAVisto does not), none of these tools support such a variety of network models as GraphCrunch does (see Table 3). Note that tYNA does not generate random models at all and it searches for subgraphs in real-world networks only. Furthermore, GraphCrunch determines the fit of the various network models to the real-world networks with respect to an array of various global and local network properties; none of the other currently available network analysis software tools have this functionality.

Out of the above mentioned software packages that generate random graphs corresponding to the real-world networks, only mfinder can keep the generated random graphs after the output is created, but not by default. Pajek is capable of generating random networks, but not for the purpose of comparing them with the data. In contrast, GraphCrunch creates, compares with the data, and outputs all of the random graphs that it generates. While some of the software packages that compute global network properties provide only simple statistics (e.g., averages) without outputting the actual data used for computing them, GraphCrunch provides such raw data; this enables the users to perform additional analyses without having to do any further computing on the networks being analyzed.

Mfinder and FANMOD both include an option of using random subgraph sampling heuristics for speeding up the computing time, a feature that GraphCrunch currently does not support (see Section "Future directions"). However, GraphCrunch's exhaustive graphlet counting is very competitive. We tested mfinder, FANMOD, and GraphCrunch on the E. coli transcriptional regulation network [4] (we ignored the directionality of this network) on a 3.0 GHz Xenon processor with 1 GB of RAM. Using the exhaustive search mode for undirected subgraphs of size 5, mfinder spent 8 minutes 50 seconds counting, FANMOD spent 7 seconds, and GraphCrunch spent a second.

Furthermore, GraphCrunch has distributed computing capabilities for running the graphlet-based analyses in parallel over a user specified cluster of machines, a feature that is not supported by Mfinder, FANMOD, MAVisto, tYNA, or pajek. This functionality will become crucial as the biological network data sets grow.

### Future directions

Due to an increasing volume of biological network data that is becoming available, new models for representing these networks and new techniques for analyzing and comparing them will unquestionably be needed. Thus, we will keep extending GraphCrunch with new functions. One of the first extensions will be enabling GraphCrunch to deal with directed and weighted networks. Also, we will extend it to support additional network models and properties.

At the moment, GraphCrunch compares a real-world network with a series of network models with respect to network properties. We will upgrade GraphCrunch with a capability for comparing two or more real-world networks.

We will also implement two sampling-based heuristic approaches for estimating graphlet frequency distributions in PPI networks: *Targeted Node Processing *(TNP) and *Neighborhood Local Search *(NLS) [30]. TNP achieves accurate graphlet frequency distribution estimates, 300–690 times faster than the exhaustive searches. NLS performs 95–377 times faster than the exhaustive searches. We will also extend these heuristics to produce fast approximate GDDs and implement these extensions in GraphCrunch. Due to their high efficiency, these heuristics will be crucial as biological network data sets become larger and more complete.

## Conclusion

GraphCrunch is a freely available, open-source software tool that implements the latest research in analyzing and modeling of biological networks. GraphCrunch analyzes large biological and other real-world networks and finds best-fitting network models by comparing real-world networks against random graph models with respect to global and local network properties. GraphCrunch supports a variety of network models and properties. Moreover, it is easily extendible to include additional network models and properties. GraphCrunch has built-in parallel computing capabilities, a feature that will become crucial as biological network data sets grow.

As biological data become larger and more complete, the need for improving the network analysis, modeling, and comparison techniques will continue to rise. We will keep including into GraphCrunch the latest research results in network analysis and modeling. Furthermore, our longer-term goal is to make GraphCrunch capable of performing biological network alignments.

## Availability and requirements

• Project name: GraphCrunch.

• Project home page: .

• Operating systems: Linux, MacOS, and Windows Cygwin.

• Programming language: C++, Perl, and Bourne Shell scripts.

• Other requirements: Windows Cygwin version requires the LEDA 5.0.1 Cygwin license and the gcc 3.4 compiler. We recommend that Perl 5.6+ as well as dialog 0.3+ or Xdialog are also installed for each of the three operating systems.

• License: None.

• Any restrictions to use by non-academics: None.

## Authors' contributions

NP designed and implemented the routines of GraphCrunch. JL designed and implemented the integrated software package that includes these routines. TM designed the user interfaces and performed most of the software testing and documentation writing. All authors read and approved the final manuscript.

## Supplementary Material

Additional file 1 Supplementary material. The data provides information about how to install GraphCrunch and use various GraphCrunch interfaces, as well as how to interpret its output. Click here for file
