# Supplementary material for: GraphCrunch: A tool for large network analyses
Source: BMC Bioinformatics. 2008 Jan 30;9:70. doi: 10.1186/1471-2105-9-70 (PMC2275247; doi:10.1186/1471-2105-9-70)
Supplement: Additional file 1 — Supplementary material. The data provides information about how to install GraphCrunch and use various GraphCrunch interfaces, as well as how to interpret its output. [file 1471-2105-9-70-S1.pdf]

Supplementary Material for: GraphCrunch: a tool for large network analyses

Tijana Milenković , Jason Lai, and Nataša Pržulj*

Department of Computer Science, University of California, Irvine, CA 92697-3435, USA

Email: TM - tmilenko@ics.uci.edu; JL - jmlai@uci.edu; NP*- natasha@ics.uci.edu;

*Corresponding author

1 Installation

GraphCrunch runs under Linux, MacOS, and Windows Cygwin. The versions for Linux and MacOS are statically compiled and thus are ready to use after unpacking. Due to the licensing issues, the Windows Cygwin version requires the LEDA 5.0.1 Cygwin license and the gcc 3.4 compiler; the compiling instructions are available from the GraphCrunch web page given above. Note that if you intend to make modifications to the Linux or MacOS versions of GraphCrunch, you will need the LEDA licenses for these operating systems in order to compile your changes.

We recommend that Perl 5.6+ as well as dialog 0.3+ or Xdialog are also installed for each of the three operating systems. The system needs to have up to 20MB of disk space available (depending on the operating system) for installing GraphCrunch. Storing a single network takes about 600 KB of disk space, and thus processing a large number of model networks may put a demand on the available disk space in the system. Therefore, we recommend that you select to process up to 30 model networks per random graph model; note that processing a larger number of networks per random graph model is not necessary, since model networks of the same size that belong to the same random graph model and are generated using the same set of parameters (as done by GraphCrunch) have very similar network properties.

Unpacking the GraphCrunch compressed archive files (available from the "Download" section of the GraphCrunch web page) results in the directory structure presented in Table S1. This organization allows for easy extendibility of the software to include additional network models and measures. For example, to add a new model, the user only needs to put a model generator program in the *src/* directory and to place

the script that runs the new program in the *scripts/* directory. The same flexibility applies to the addition of new network properties.

2 Running GraphCrunch

2.1 The command-line interface

The command-line interface is started by the command:

```
./crunch -f graph_filename [-m model] [-p parameter] [-c comparison]
[-o output_filename] [-n num_random_graphs] [-r machine]
```

where text in square brackets is optional, but can also be repeated (see below). The syntax for specifying network models in part `-m model` of the command are `er`, `er_dd`, `geo`, `sf`, and `sticky`, corresponding to ER, ER-DD, GEO-3D, SF-BA, and STICKY, respectively. Note that the default geometric random graphs are 3-dimensional (`geo` or `geo:3d`), but a user may select any dimensionality; for example, 4-dimensional geometric random graphs are generated by: `-m geo:4d`. In the case of multiple models, parameters, comparisons or machines, multiple switches (e.g., `-m er -m sf`) or strings (e.g., `-m "er sf"`) can be used. Network properties in GraphCrunch are subdivided into *parameters* (denoted by `-p` in the above command) and *comparisons* (denoted by `-c` in the command). The "parameters" denote network properties computed on both the data and model networks: the average clustering coefficient (denoted by `clustcoef-avg`), the average diameter (denoted by `diameter-avg`), and the graphlet count, i.e., the total number of graphlets in the network (denoted by `graphlet-count`). The average diameters and average clustering coefficients of model networks can be *compared* to those of a real-world network (i.e., can be used as "comparisons") in two ways: (1) `-c diameter-avg:difference` and `-c clustcoef-avg:difference` calculate the absolute values of their differences; and (2) `-c diameter-avg:percenterr` and `-c clustcoef-avg:percenterr` compute their percentage differences. "Comparisons" in GraphCrunch are properties of a real-world network that are *compared* against those of model networks; they include the degree distribution (denoted by `degree-distrib`), the clustering spectrum (denoted by `clust-spectrum`), the spectrum of shortest path lengths (denoted by `diameter-spectrum`), the GDD-agreement (denoted by `gdd-agreement:amean` for arithmetic mean, and `gdd-agreement:gmean` for geometric mean), and the RGF-distance (denoted by `graphlet-dist`). The degree distributions, the clustering spectra, and the shortest path length spectra of two networks are compared by the Pearson's correlation. RGF-distances and GDD-agreements are calculated as in [1] and [2], respectively.

Since finding local structural properties of networks is compute intensive, we have enabled GraphCrunch with parallel computing capabilities. A user can distribute the GraphCrunch processing over a cluster of machines, by including -r "machine1 machine2 machineN" in the above command, where machine1, machine2 and machineN are machine names.

2.2 The run-dialog interface

The run-dialog interface is started by the command: ./run-dialog <input_graph_name>. The check-boxes are used to (un)select the following: the random graph models (Figure S3 A), the number of networks per random graph model (Figure S3 B), network parameters (Figure S3 C) and comparisons (Figure S3 D), and the name of the output file (Figure S3 E). The processing with the specified selections can either start immediately (by choosing "*Done — Run crunch*" option in Figure S3 F), or "advanced options" can be configured to change network models, parameters, comparisons, or the machines over which to distribute the processing (see GraphCrunch webpage for details).

2.3 GraphCrunch on-line web user interface

The GraphCrunch on-line web user interface is available from the GraphCrunch webpage. To ensure a fair access for all users to our computing resources, the maximum network size is limited to 20,000 nodes and 80,000 edges, the maximum number of random graphs per network model is limited to five, and a user is allowed to process up to four real-world networks per day. (No limitations exist for the other two GraphCrunch interfaces that users run on their own machines.)

From the main on-line GraphCrunch web page, users can login to their accounts, or access the help page and see some examples. New users are given temporary "guest" accounts that are intended for short-term use. At any time, users can change their guest accounts into permanent ones that are password protected. Both types of accounts allow access to the on-line GraphCrunch functions and the users' data sets at any time.

After login, the menu of the on-line GraphCrunch is organized as follows:

- The *Data Sets* page (Figure S4) allows for uploading the new data sets and accessing already submitted data sets. If the data sets are still being processed, users can monitor the status of their processing. For a given data set, a user can download the input file and the results. The input file is available for download in three different formats: edge list format (*.txt*), LEDA format (*.gw*) and GML format (*.gml*). The results are available in *.xls* format.

- The *Results* page (Figure S5) provides visualization of the results of the processed data sets. A user can choose to visualize all or some of his or her data sets. Spectral properties are plotted: the degree distribution, the spectrum of shortest path lengths (denoted by "distance spectrum"), the clustering spectrum, and the graphlet frequency spectrum. A "mouse over" these plots gives the Pearson's correlation coefficients between the properties of the data and the model networks. Other parameters and comparisons are given as numerics.
- The *GDD Viewer* page (Figure S6) presents plots of graphlet degree distributions (GDDs) for each of the 73 orbits of real-world and model networks. GDDs of only one model network for the corresponding random graph model are displayed.
- The *My Account* page enables users to manage their accounts.
- The *Help* page provides instructions on how to use the on-line GraphCrunch and how to interpret the results.

2.4 Interpreting results

An example of the *tabular output* file is presented in Table S2: network "*Net1.gw*" is compared against five network models, three random networks per network model are used, and the comparisons are done with respect to all of the network properties currently supported by GraphCrunch. The command that produces these results is:

```
./crunch -f Net1.gw -p "diameter-avg clustcoef-avg graphlet-count" -c
"degree-distrib diameter-spectrum clust-spectrum
gdd-agreement:amean gdd-agreement:gmean graphlet-dist"
-m "er er_dd geo sf sticky" -n 3 -o output_example.tsv
```

The *set of intermediate files* is stored in the subdirectories of the *data/<input-file-name>* directory (e.g., the generated model networks that correspond to the input network, are saved in the subdirectory *data/<input-file-name>/models*).

The *visualized output*, i.e., the plots and all of the files necessary for their generation (e.g., *.gnuplot* files) are stored in the *plots/* directory. Before executing the plot function of GraphCrunch (given below), the following must be satisfied: (1) the data sets must be processed with *./crunch* command (described in Section 2.1) and the corresponding intermediate files must exist in the *data/* directory; and (2) *gnuplot*

command-line plotting utility must be available on a user's system. Visualized output files are created by running the *plot.sh* script (found in the *contrib/* directory); the syntax for running this script is:

```
./contrib/plot.sh -d input-network -m model -c comparison -o output-file
```

where multiple input networks or models may be specified, as illustrated in the example below. Examples of plots are presented in Figure S7: four network properties for three input data sets and five network models per data set are illustrated. The command used to produce Figure S7 A is:

```
./contrib/plot.sh -d Net1 -d Net2 -d Net3 -m er -m er_dd -m geo -m sf -m sticky -c
gdd-agreement:amean -o plot_ex_gdd
```

References

1. Pržulj N, Corneil DG, Jurisica I: **Modeling Interactome: Scale-Free or Geometric?** *Bioinformatics* 2004, **20**(18):3508–3515.
2. Pržulj N: **Biological Network Comparison Using Graphlet Degree Distribution**. *Bioinformatics* 2006, **23**:e177–e183.

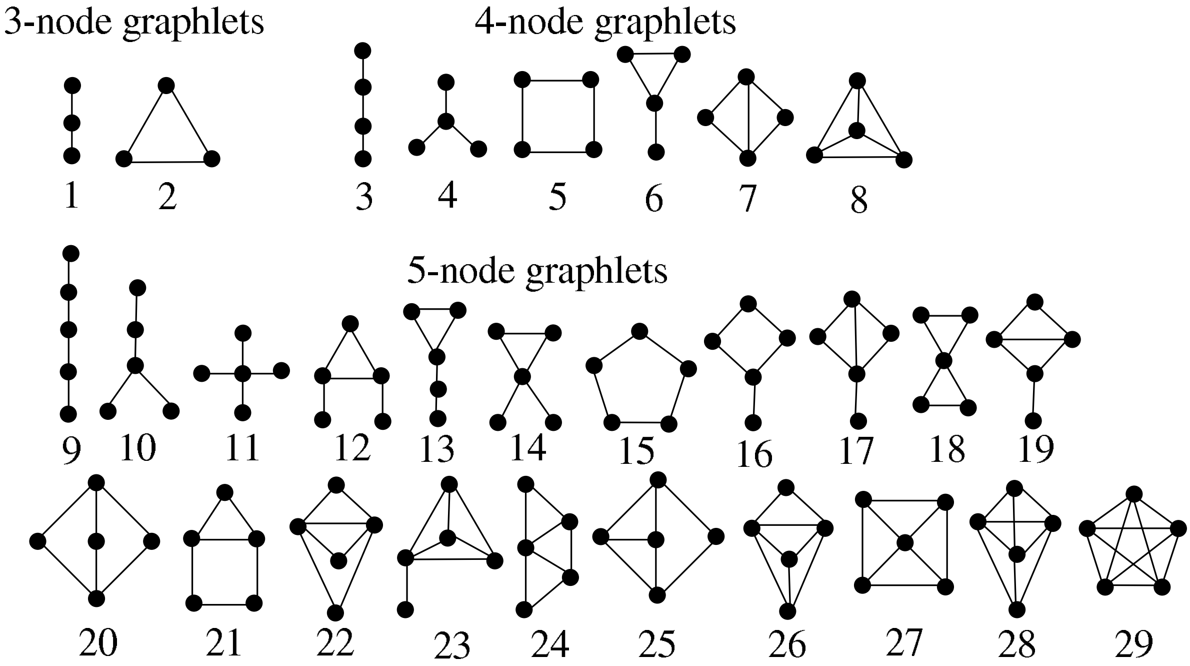

Figure S1. All 3-node, 4-node and 5-node graphlets [1].

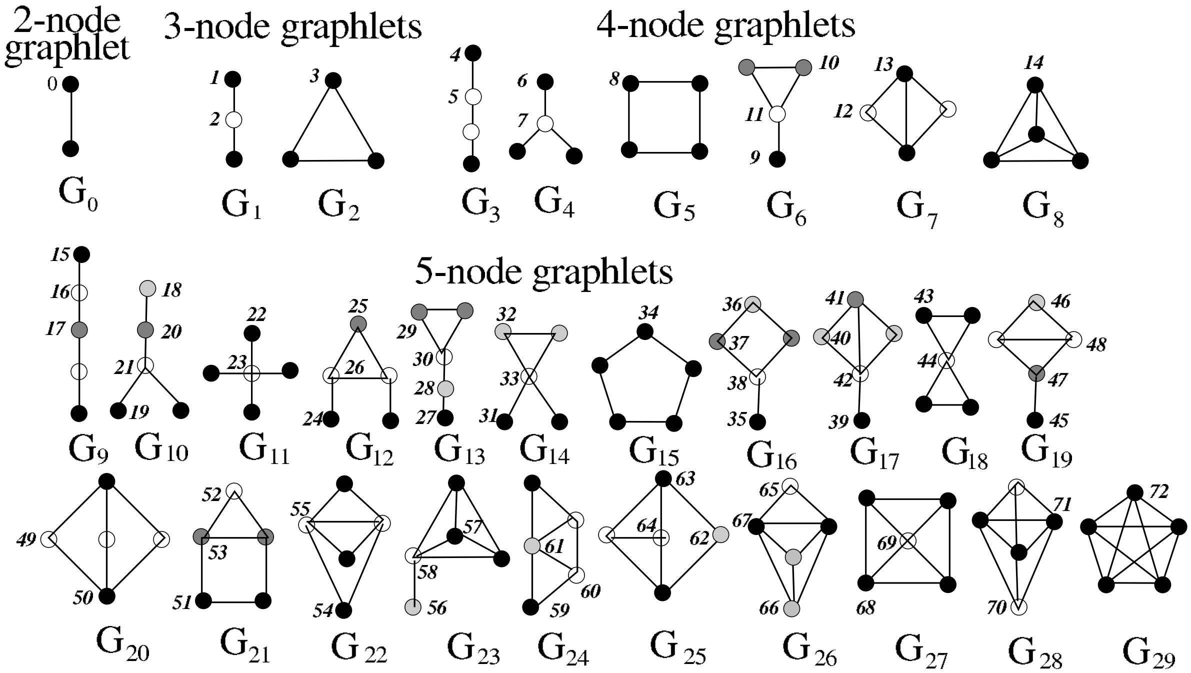

Figure S2. Automorphism orbits 0, 1, 2,..., 72 for the thirty 2-, 3-, 4-, and 5-node graphlets G_0, G_1,..., G_{29}. In a graphlet G_i, $i \in$ 0, 1,..., 29, nodes belonging to the same orbit are of the same shade [2].

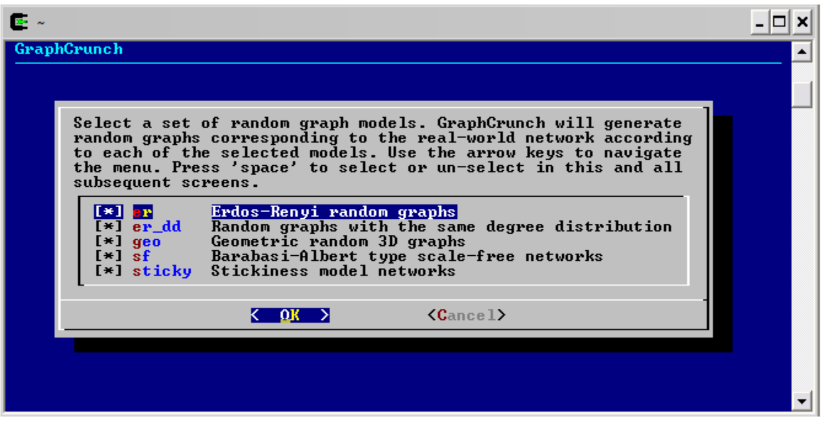
GraphCrunch
Select a set of random graph models. GraphCrunch will generate random graphs corresponding to the real-world network according to each of the selected models. Use the arrow keys to navigate the menu. Press 'space' to select or un-select in this and all subsequent screens.
[*] er Erdos-Renyi random graphs
[*] er_dd Random graphs with the same degree distribution
[*] geo Geometric random 3D graphs
[*] sf Barabasi-Albert type scale-free networks
[*] sticky Stickiness model networks
< OK >
<Cancel>
(A)

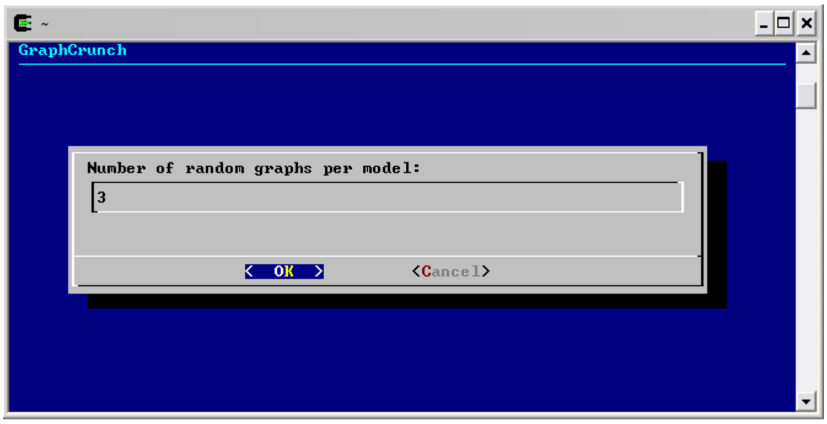
GraphCrunch
Number of random graphs per model:
3
< OK >
<Cancel>
(B)

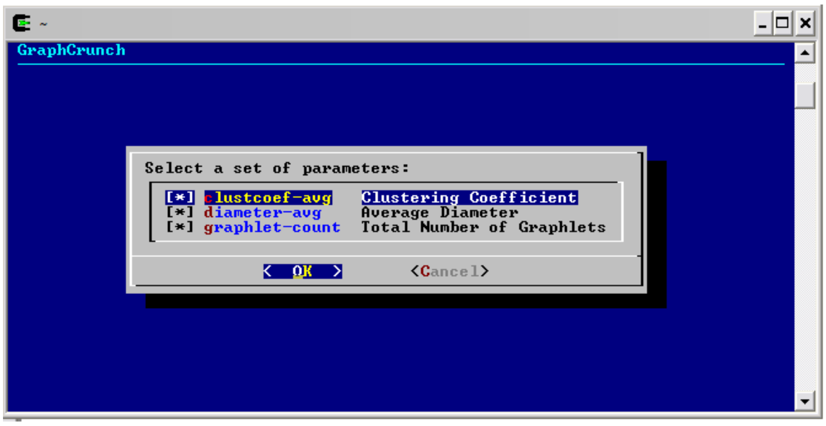
GraphCrunch
Select a set of parameters:
[*] clustcoef-avg Clustering Coefficient
[*] diameter-avg Average Diameter
[*] graphlet-count Total Number of Graphlets
< OK >
<Cancel>
(C)

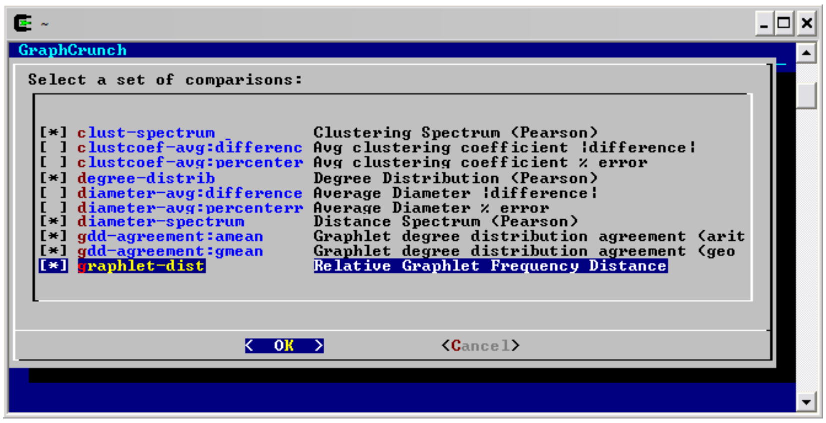
GraphCrunch
Select a set of comparisons:
[*] clust-spectrum Clustering Spectrum (Pearson)
[ ] clustcoef-avg:differenc Avg clustering coefficient |difference|
[ ] clustcoef-avg:percenter Avg clustering coefficient % error
[*] degree-distrib Degree Distribution (Pearson)
[ ] diameter-avg:difference Average Diameter |difference|
[ ] diameter-avg:percenterr Average Diameter % error
[*] diameter-spectrum Distance Spectrum (Pearson)
[*] gdd-agreement:amean Graphlet degree distribution agreement (arit
[*] gdd-agreement:gmean Graphlet degree distribution agreement (geo
[*] graphlet-dist Relative Graphlet Frequency Distance
< OK >
<Cancel>
(D)

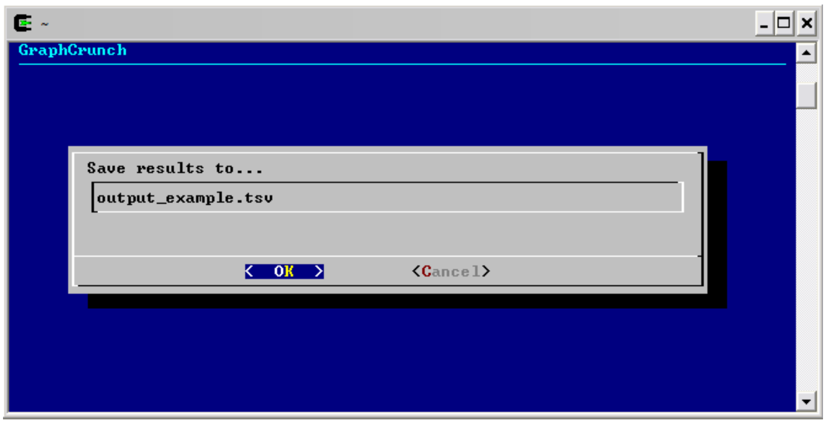

(E)

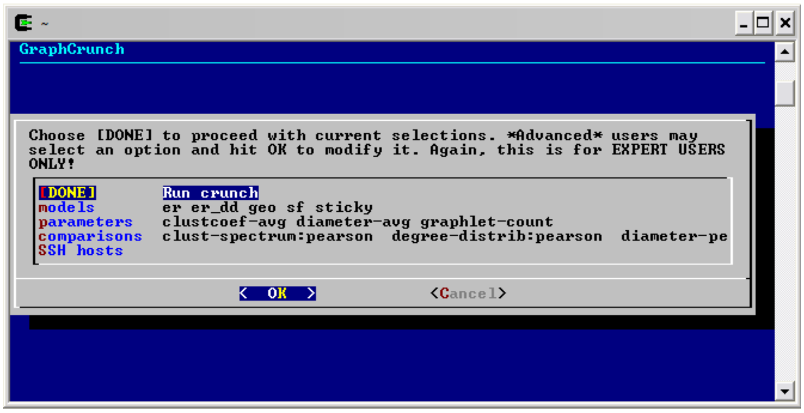

(F)

Figure S3. The sequence of steps in the run-dialog GraphCrunch interface: (A) choosing network models against which the real-world network is to be compared; in the figure, all five network models (er, er_dd, geo, sf, and sticky) are selected; (B) specifying the number of random graphs to be generated per network model; in the figure, 3 graphs are to be generated per random network model; (C) choosing the parameters (as described in Section 2.1) to be computed for the data and model networks; in the figure, all three parameters (clustcoef-avg, diameter-avg, and graphlet-count) have been selected; (D) choosing the comparisons (as described in Section 2.1) to be computed between the data and model networks; in the figure, the following comparisons have been selected: clust-spectrum, degree-distrib, diameter-spectrum, gdd-agreement:amean, gdd-agreement:gmean, and graphlet-dist; (E) specifying the name of the tabular output file; in the figure, the file named “output example.tsv” is designated as the output file; (F) proceeding with processing with the current selections (by choosing the *“Done — Run crunch”* option).

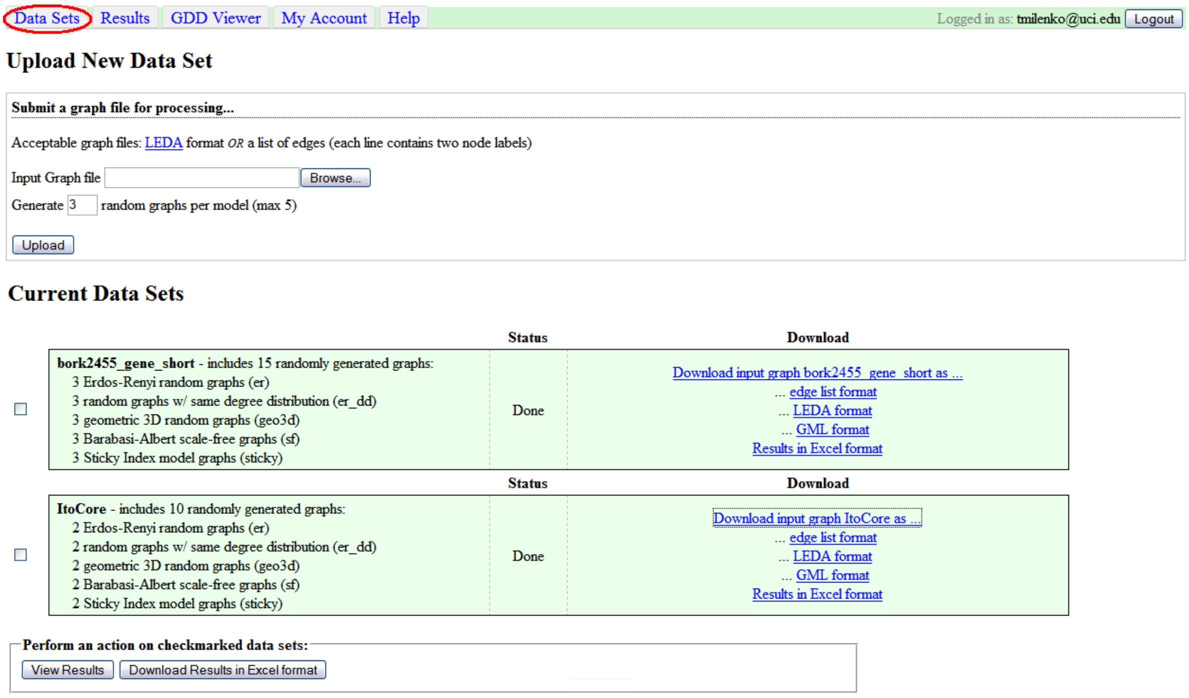

Figure S4. *Data sets* page of the GraphCrunch on-line web user interface. Users can upload the new data sets (in the “Upload New Data Sets” section of the page) and access already submitted data sets (in the “Current Data Sets” section of the page). If the data sets are still being processed, users can monitor the status of the processing. For a given data set, a user can download the input file (three formats are available) and the results.

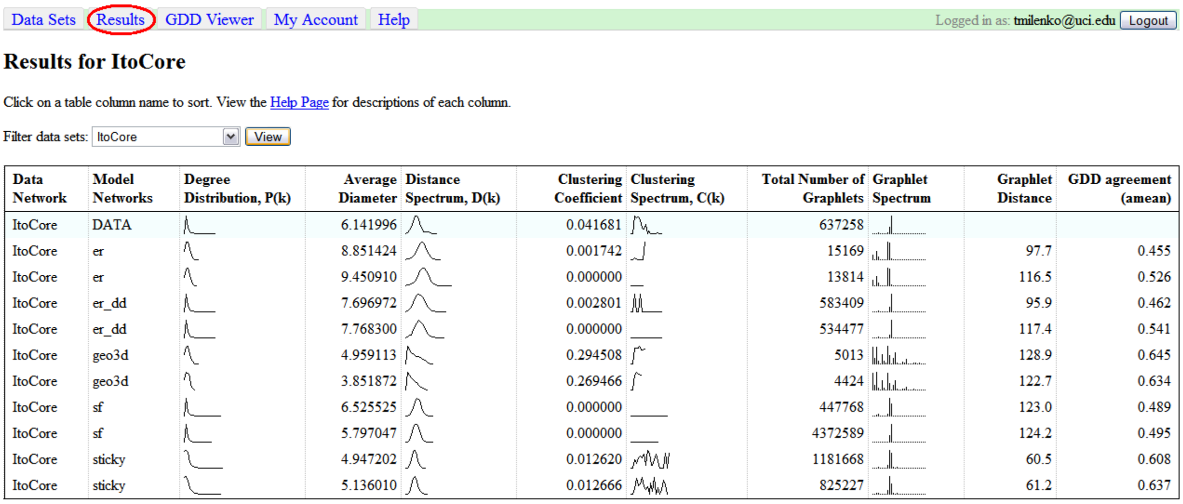

Data Network	Model Networks	Degree Distribution, P(k)	Average Diameter	Distance Spectrum, D(k)	Clustering Coefficient	Clustering Spectrum, C(k)	Total Number of Graphlets	Graphlet Spectrum	Graphlet Distance	GDD agreement (amean)
ItoCore	DATA		6.141996		0.041681		637258			
ItoCore	er		8.851424		0.001742		15169		97.7	0.455
ItoCore	er		9.450910		0.000000		13814		116.5	0.526
ItoCore	er_dd		7.696972		0.002801		583409		95.9	0.462
ItoCore	er_dd		7.768300		0.000000		534477		117.4	0.541
ItoCore	geo3d		4.959113		0.294508		5013		128.9	0.645
ItoCore	geo3d		3.851872		0.269466		4424		122.7	0.634
ItoCore	sf		6.525525		0.000000		447768		123.0	0.489
ItoCore	sf		5.797047		0.000000		4372589		124.2	0.495
ItoCore	sticky		4.947202		0.012620		1181668		60.5	0.608
ItoCore	sticky		5.136010		0.012666		825227		61.2	0.637

Figure S5. *Results* page of the GraphCrunch on-line web user interface. It provides visualization of the results of the processed data sets, with an option for filtering the data sets for which the results will be shown. In the figure, the results for the data set named "ItoCore" (denoted by "DATA" and highlighted in blue) and five model networks (denoted by "er", "er_dd", "geo3d", "sf", and "sticky") are shown. Two networks are processed per network model. Spectral properties are plotted: the degree distribution, the spectrum of shortest path lengths (denoted by "distance spectrum"), the clustering spectrum, and the graphlet frequency spectrum. A "mouse over" these plots gives the Pearson's correlation coefficients between the properties of the data and the model networks. Other parameters (average diameter, clustering coefficient, total number of graplets) and comparisons (RGF-distance, denoted by "Graphlet Distance", and GDD-agreement) are given as numerics.

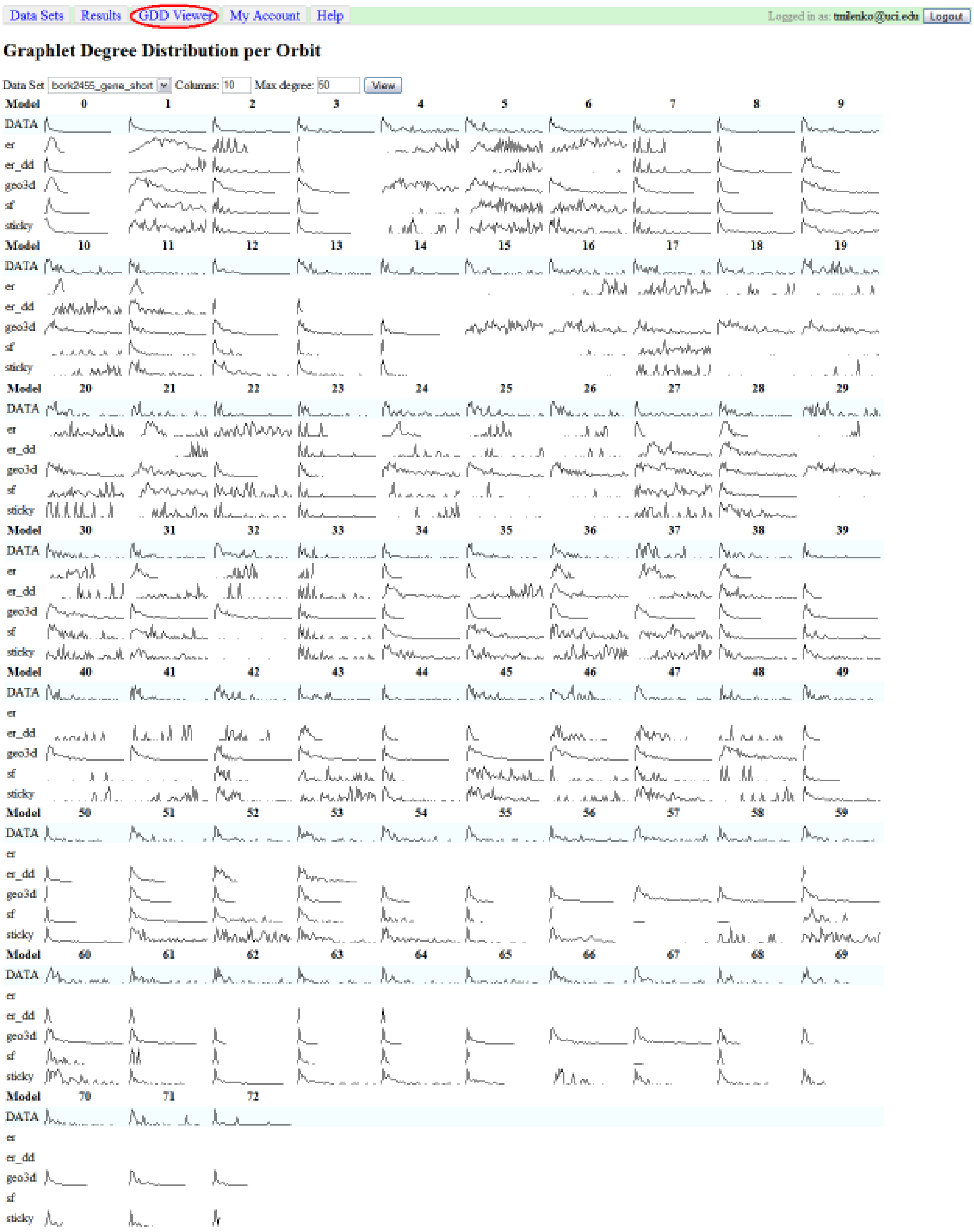

Figure S6. *GDD Viewer* page of the GraphCrunch on-line web user interface. It contains plots of graphlet degree distributions (GDDs) for each of the 73 orbits of real-world and model networks. In the figure, GDDs of the data set named "bork2455_gene_short" (denoted by "DATA" and highlighted in blue) and five model networks (denoted by "er", "er_dd", "geo3d", "sf", and "sticky") are shown. The plots illustrating ten GDDs are displayed in each row, and 50 is chosen to be the maximum degree on x-axis in each of the plots.

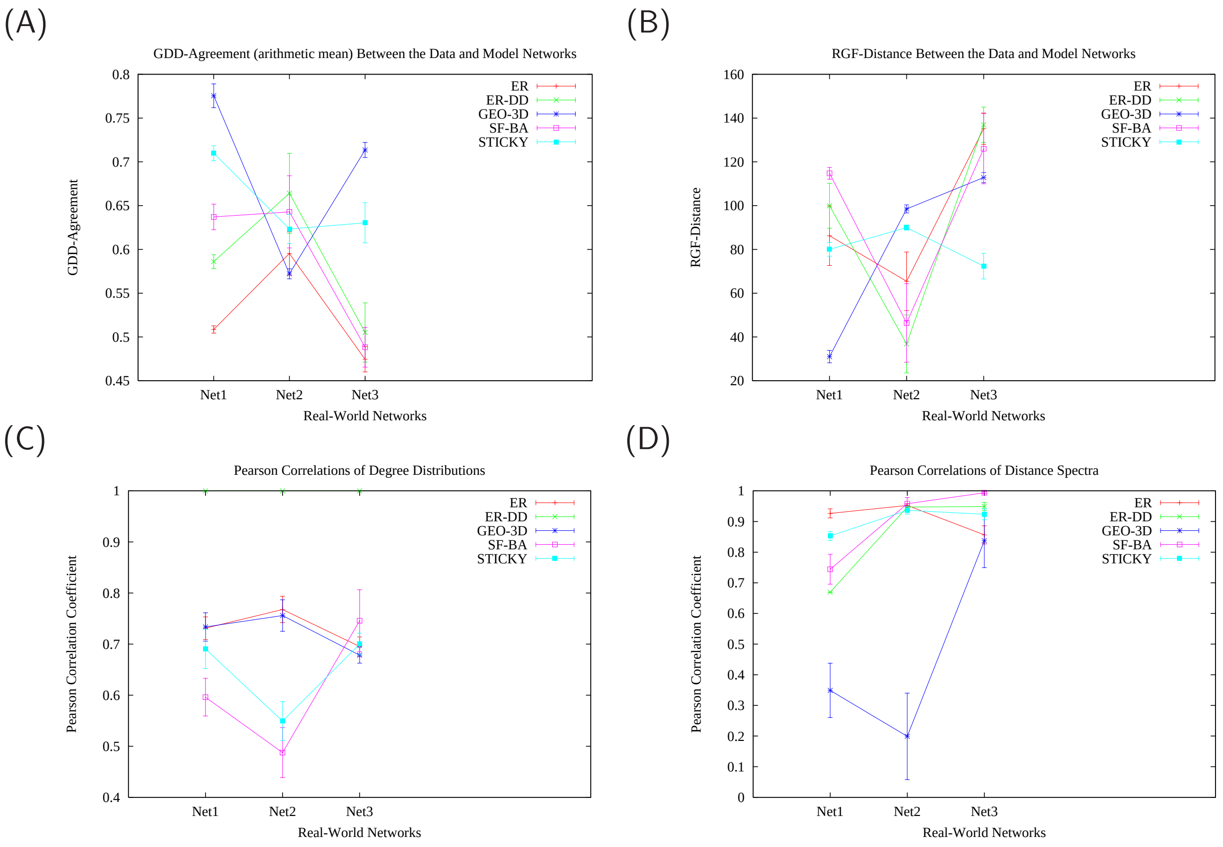

Figure S7. Examples of plots that illustrate the fit of five network models (ER, ER-DD, GEO-3D, SF-BA, and STICKY) to three data sets (Net1, Net2 and Net3) with respect to four networks properties: (A) GDD-agreement, (B) RGF-distance, (C) the degree distribution, and (D) the spectrum of shortest path lengths. Points in panels represent averages of properties over model networks belonging to the same random graph model; the error bars represent one standard deviation above and below the average.

Table S1 - The directory structure of GraphCrunch.

File or Directory:	*Content:*
./run-dialog	A text User Interface wizard for running GraphCrunch.
./crunch	A command-line tool for running GraphCrunch.
README.txt	The “readme” file with the explanations on how to use GraphCrunch.
batch/	Scripts and temporary data for remote batch processing.
contrib/	Scripts and utilities that use additional software (e.g., gnuplot or R); users can contribute with their own add-ons.
data/	Sets of intermediate files.
doc/	Advanced documentation for extending the software package and troubleshooting.
plots/	Visualized output files for the user-friendly graphical interpretations of the results.
sample_graphs/	Some sample graphs to try out.
scripts/	Scripts that use the results of network model generators and programs computing network properties to create the statistics that summarize the results.
src/	Source code (for advanced users and developers).
tmp/	Temporary data.

Data Network	Model Networks	Random Networks/Stats	Average Diameter	Clustering Coefficient	Total Number of Graphlets	Degree Distribution (Pearson)	Distance Spectrum (Pearson)	Clustering Spectrum (Pearson)	GDD Agreement (amean)	GDD Agreement (gmean)	RGF Distance
Net1	DATA		5.193916	0.343733	7084875						
Net1	er	1	4.470773	0.00377221	709093	0.749373	0.899005	0.315557	0.520145	0.485791	92.7365
Net1	er	2	4.46555	0.00414739	686221	0.729027	0.9283244	0.361904	0.487339	0.458529	99.9765
Net1	er	3	4.48149	0.00448979	683101	0.728214	0.928324	0.290587	0.506549	0.482846	69.5645
Net1	er	AVG	4.4726	0.00413646	692805	0.735538	0.918551	0.322683	0.504678	0.475722	87.4258
Net1	er	STDDEV	0.0081263	0.00035892	14191.8	0.0119884	0.016927333	0.0361885	0.0164829	0.0149622	15.8863
Net1	er_dd	1	3.654958	0.0188613	11798396	1	0.66933	0.553144	0.572865	0.5397	98.1254
Net1	er_dd	2	3.646424	0.0212761	12127887	1	0.66933	0.687245	0.608612	0.584074	126.53
Net1	er_dd	3	3.66037	0.0157781	11623933	1	0.66933	0.577731	0.599248	0.564781	93.6498
Net1	er_dd	AVG	3.65392	0.0186385	1.19E+07	1	0.66933	0.60604	0.593575	0.562852	106.102
Net1	er_dd	STDDEV	0.007031	0.00275576	255920	0	0	0.071392	0.0185364	0.0222498	17.8324
Net1	geo	1	12.459794	0.480566	197686	0.701905	0.458554	0.269989	0.770701	0.757404	31.086
Net1	geo	2	11.725866	0.475522	174527	0.723629	0.440894	0.319434	0.775507	0.764023	30.1048
Net1	geo	3	12.045253	0.471293	175465	0.721004	0.398954	0.309954	0.763752	0.751506	31.5283
Net1	geo	AVG	12.077	0.475794	182559	0.715513	0.432800667	0.299792	0.769987	0.757644	30.9064
Net1	geo	STDDEV	0.367991	0.00464247	13108.5	0.0118574	0.030613176	0.0262421	0.00590997	0.00626196	0.728553
Net1	sf	1	3.733036	0.027936	14133844	0.569115	0.781542	0.294485	0.657569	0.622761	108.738
Net1	sf	2	3.709808	0.0397339	24255912	0.635718	0.725214	0.191622	0.620613	0.596942	118.092
Net1	sf	3	3.727588	0.0426044	20284643	0.746446	0.781542	0.415984	0.640051	0.58829	117.796
Net1	sf	AVG	3.72348	0.0367581	1.96E+07	0.650426	0.762766	0.300697	0.639411	0.602664	114.875
Net1	sf	STDDEV	0.0121474	0.0077738	5.10E+06	0.0895758	0.032520986	0.11231	0.0184863	0.0179338	5.31715
Net1	sticky	1	3.534456	0.0379715	25480754	0.651102	0.834954	0.0659298	0.704925	0.684772	79.2167
Net1	sticky	2	3.533758	0.0353593	24539324	0.651161	0.821229	0.163612	0.704726	0.683138	87.0146
Net1	sticky	3	3.538667	0.0373394	26291465	0.674981	0.834954	0.160287	0.69473	0.673913	76.8051
Net1	sticky	AVG	3.53563	0.0368901	2.54E+07	0.659081	0.830379	0.129943	0.70146	0.680608	81.0121
Net1	sticky	STDDEV	0.0026558	0.00136284	876883	0.0137695	0.007924132	0.0554619	0.00582949	0.00585503	5.33631

Table S2

An example of the output file resulting from processing input network *Net1.gw* by GraphCrunch. The five network models and all of the currently supported properties are presented. Three networks per random graph model were generated (denoted by 1, 2, and 3 in column "*Random Networks/Stats*"). Thus, the total number of random networks analyzed in this example is 3x5=15. The column denoted by "*Data Network*" contains the name of the real-world network being analyzed. The column denoted by "*Model Networks*" contains the names of network models against which the data is being compared ("er", "er_dd", "sf", "geo", and "sticky"). Random graphs from the same network model are denoted by a sequence of integers presented in column "*Random Network/Stats*"; in the same column, "AVG" and "STDDEV" denote that the fields in these rows contain the averages and standard deviations of network properties (given in columns to the right) computed over all random graphs from the given network model (that were generated and analyzed by GraphCrunch). The columns denoted by "*Average Diameter*" and "*Clustering Coefficient*" contain the average diameter and the average clustering coefficient of a network, respectively. The column denoted by "*Total Number of Graphlets*" contains the total number of all 2-5-node graphlets in a network. The columns denoted by "*Degree Distribution (Pearson)*", "*Distance Spectrum (Pearson)*" and "*Clustering Spectrum (Pearson)*" contain the Pearson's rank correlation coefficients of the degree distributions, the spectra of shortest path lengths and the clustering spectra between the real-world and model networks, respectively. The columns denoted by "*GDD agreement (amean)*" and "*GDD agreement (gmean)*" contain the arithmetic and geometric means of GDD-agreements between the real-world and model networks, respectively. Finally, the column denoted by "*RGF Distance*" contains the RGF-distance between the data and model networks.

Table S3 - Comparison of the running times t_{ER}, t_{GEO-3D}, and t_{SF-BA} of GraphCrunch for five input networks $G(|V|,|E|)$ (where $|V|$ is the number of nodes and $|E|$ is the number of edges in network G) of different sizes, but with constant edge densities, originating from three different network models, ER, GEO-3D, and SF-BA, respectively. We report the total number of CPU time, in seconds, that each GraphCrunch run used.

$G(\|V\|,\|E\|)$	G(100,294)	G(500,1471)	G(1000,2941)	G(2000,5882)	G(3000,8820)	G(4000,11764)	G(5000,14706)	G(7500,22050)	G(10000,29411)
t_{ER}	7.772	28.589	61.255	271.844	856.625	1907.959	3445.887	7076.198	12807.384
t_{GEO-3D}	7.792	30.277	75.764	256.368	595.137	1868.588	3078.024	5839.688	12894.561
t_{SF-BA}	8.264	44.522	158.657	534.077	1354.412	4301.324	8518.000	14279.14	48138.136

Table S4 - Comparison of the running times t_{ER}, t_{GEO-3D}, and t_{SF-BA} of GraphCrunch for five input networks $G(|V|,|E|)$ (where $|V|$ is the number of nodes and $|E|$ is the number of edges in network G) with same number of nodes, but different edge densities, originating from three different network models, ER, GEO-3D, and SF-BA, respectively. We report total number of CPU time, in seconds, that each GraphCrunch run used.

$G(\|V\|,\|E\|)$	G(1000,2273)	G(1000,2941)	G(1000,4167)	G(1000,7143)	G(1000,11100)
t_{ER}	30.929	61.255	131.952	600.385	2369.084
t_{GEO-3D}	49.719	75.764	128.964	600.597	2335.725
t_{SF-BA}	69.896	158.657	292.89	1464.963	4869.388
